# Integrative Phosphoproteomic Characterization of ULK1 Phosphosites Reveals Co-Regulatory Networks in Autophagy and Metabolic Adaptation

**DOI:** 10.3390/ijms27198892

**Published:** 2026-10-07

**Authors:** Furqaan Ahmed Basha, Sreeshma Ravindran Kammarambath, Leona Dcunha, Althaf Mahin, Athira Perunelly Gopalakrishnan, Alimath Sambreena, Inamul Hasan Madar, Rajesh Raju

**Affiliations:** Centre for Integrative Omics Data Science (CIODS), Yenepoya (Deemed to be University), Mangalore 575018, Karnataka, India; furqaanahmed29@gmail.com (F.A.B.); sreeshmarkk@gmail.com (S.R.K.); leonadcunha@gmail.com (L.D.); althafmahin99@gmail.com (A.M.); 19228@yenepoya.edu.in (A.P.G.); sambreenaibrahim75@gmail.com (A.S.); oncoinam@gmail.com (I.H.M.)

**Keywords:** ULK1, autophagy, phospho-regulatory network, metabolic enzymes, lipolysis

## Abstract

Unc-51-like autophagy-activating kinase 1 (ULK1), a serine/threonine kinase, is the central regulator of autophagy initiation and plays a crucial role in cellular homeostasis, metabolic adaptation, and stress responses. We performed a large-scale phosphoproteomic data analysis of ULK1 by systematically integrating and analyzing 891 profiling and 245 differential human phosphoproteomic datasets. S450, S623, and S638 emerged as the predominant phosphosites in ULK1, with the highest frequency of detection across multiple cellular phosphoproteomics datasets. Conservation analysis showed that these phosphosites are conserved across mammalian species and human ULK1 and ULK2 (Unc-51-like autophagy-activating kinase 2). The co-regulatory networks associated with these phosphosites, including interacting proteins, upstream kinases, and downstream substrates, were systematically analyzed. The phosphosite co-regulated ULK1 interactome revealed a network of associations linking ubiquitin-dependent cargo recognition to autophagosome biogenesis, trafficking, and maturation, indicating that ULK1 phosphoregulation may extend beyond its established role in autophagy initiation and be associated with multiple stages of selective autophagy. Notably, ULK1 phosphosites were linked to key metabolic enzymes in glycogen, sphingolipid, phospholipid, and glycolytic pathways, as well as classical lipolysis. Protein-normalized analysis across 12 cancer cohorts showed significant upregulation of the predominant ULK1 phosphosites, most frequently at S450 and S623. Comparative analysis with an independent ULK1/2 knockout phosphoproteomic dataset further supported the relevance of these identified co-regulated protein phosphosites. Collectively, this study provides a phosphosite-resolved view of ULK1 regulation and identifies candidate phosphosites and associated proteins for future functional and mechanistic studies.

## 1. Introduction

Unc-51-like autophagy-activating kinase 1 (ULK1) is a serine/threonine kinase and the primary mammalian homolog of the yeast autophagy-initiating kinase ATG1 [1]. The human ULK1 gene is located on chromosome 12q24.33 and encodes a 1050-amino-acid protein [2]. It is a key regulator of autophagy initiation, a highly conserved cellular process that maintains cellular homeostasis by degrading and recycling intracellular pathogens, misfolded proteins, and damaged organelles such as the endoplasmic reticulum, mitochondria, and peroxisomes, which are then degraded through the lysosomal pathway [3]. ULK1 belongs to the ULK kinase family, which comprises five mammalian members: ULK1, ULK2, ULK3, ULK4, and STK36. Among these, ULK1 and ULK2 are the principal regulators of autophagy, and they share approximately 52% overall sequence identity and 78% sequence identity within their kinase domains. Both function as mammalian orthologues of the yeast ATG1 kinase and play central roles in autophagy initiation [4]. In contrast, the remaining family members, ULK3, ULK4, and STK36, serve non-canonical developmental and signaling functions outside of conventional autophagy [5].

Structurally, ULK1 has three main regions: an N-terminal kinase domain for its catalytic activity, a central serine/proline-rich (SPR) region with many regulatory phosphorylation sites, and a C-terminal domain that mediates interactions with the core autophagy machinery. ULK1 activity is primarily controlled through phosphorylation by two opposing upstream kinases. Under nutrient-rich conditions, mechanistic target of rapamycin complex 1 (mTORC1) phosphorylates ULK1 at Ser638 and Ser758 in human ULK1 (UniProt O75385), corresponding to Ser637 and Ser757 as reported in the mouse Ulk1 studies (UniProt O70405), inhibiting its kinase activity and modulating its interaction with AMPK, thereby suppressing autophagy induction. During energy deprivation, AMPK inhibits mTORC1 while directly phosphorylating and activating ULK1, together promoting autophagy induction [6]. By integrating these opposing signals, ULK1 acts as a central signaling hub that coordinates cellular energy status with autophagic flux [7]. Through its C-terminal domain, ULK1 interacts with FIP200, ATG13, and ATG101, which are core components of the ULK1 complex that initiates autophagy [8,9]. After initiation, ULK1 undergoes autophosphorylation and phosphorylates ATG13 and FIP200, which increases the activity of the ULK1 complex at endoplasmic reticulum-associated membrane sites, where phagophore formation begins.

Beyond its canonical role in autophagy initiation, ULK1 is involved in both bulk macroautophagy and selective autophagy processes, including mitophagy and lipophagy, highlighting its broad significance in maintaining cellular homeostasis in both normal and disease conditions [10]. Dysregulation of ULK1 activity and its phosphorylation-dependent regulation have been associated with impaired autophagic flux, metabolic dysfunction, and cancer-associated phenotypes; however, a comprehensive phosphoproteomic characterization of its site-specific regulatory network across large-scale human datasets has yet to be explored, which highlights the importance of systematically investigating its phosphosite-specific regulatory landscape [11].

Integrating publicly available large-scale phosphoproteomic datasets from diverse biological contexts has become an effective strategy for systematically identifying functionally relevant phosphosites and exploring their biological roles through co-regulation analysis, GO/pathway enrichment, mapping of interactors and prediction of substrates [12]. Building on this integrative phosphoproteomic approach, we aimed to identify and characterize the predominant phosphosites of ULK1 and explore their potential functional significance. We systematically defined the co-regulatory landscape of ULK1 phosphosites S450, S623, and S638, identifying associated interactors, kinases, substrates, and metabolic enzymes linked to autophagy, vesicle trafficking, and energy signaling.

## 2. Results

### 2.1. Assembly of Phosphoproteomic Datasets and Conservation Analysis of Predominant ULK1 Phosphosites

To identify phosphorylation patterns that may be functionally relevant to ULK1, we screened publicly available cellular phosphoproteomics studies and identified 891 profiling datasets and 245 differential regulation datasets, with detailed data provided in Supplementary Materials File S1. These datasets included Class I ULK1 phosphosites, with 57 and 43 mapped phosphosites identified in the profiling and differential regulation datasets, respectively. Among the detected phosphosites in ULK1, S450, S623, and S638 were identified as the predominant phosphosites with the highest frequency of detection across the curated datasets. Specifically, S450, S623, and S638 were detected in 619, 674, and 583 profiling datasets and 77, 70, and 61 differential datasets, respectively. Taken together, these three phosphosites represented approximately 93% and 69% of the phosphorylation events in the profiling and differential regulation datasets, respectively. A lollipop plot of ULK1 phosphosites with their detection frequencies in global cellular phosphoproteomics datasets is illustrated in Figure 1. Previously, Gopalakrishnan et al. (2025) had classified human kinases based on the positional distribution of their predominant phosphorylation sites relative to the kinase domain into categories such as PiKD (predominant site within the kinase domain) and PoKD (predominant site outside the kinase domain) [13]. According to this classification, the predominant ULK1 phosphosites S450, S623, and S638 are located outside the kinase domain, classifying ULK1 as a PoKD kinase.

To provide functional support for the predominant ULK1 phosphosites, we examined the conservation of S450, S623, and S638 among human ULK family members and across mammalian species. We further compared the predominant ULK1 phosphosites with the corresponding regions in the human ULK family member, consisting of ULK1, ULK2, ULK3, ULK4, and STK36. ULK1 and ULK2 are well established as key regulators of autophagy initiation, functioning within the ULK1/2-ATG13-FIP200 complex, whereas ULK3, ULK4, and STK36 perform distinct, non-canonical functions that extend beyond core autophagy regulation. Alignment showed that S450 and S623 are conserved between ULK1 and ULK2. In contrast, the residue aligning with S638 in ULK2 is an alanine rather than a serine, indicating that phosphorylation at this position is specific to ULK1. ULK3, ULK4, and STK36 showed substantial sequence divergence at these positions, consistent with their distinct functional roles outside the autophagy-initiating ULK1/ULK2 subfamily. Multiple sequence alignment of ULK1 orthologs from human (*Homo sapiens*), mouse (*Mus musculus*), rat (*Rattus norvegicus*), rabbit (*Oryctolagus cuniculus*), Eurasian red squirrel (*Sciurus vulgaris*), small-eared galago (*Otolemur garnettii*), pig (*Sus scrofa*), goat (*Capra hircus*), dog (*Canis lupus familiaris*), and rhesus macaque (*Macaca mulatta*) showed that S450, S623, and S638 are conserved across these species, indicating that these residues have been conserved throughout mammalian evolution and may play important functional roles. Together, these findings provide conservation-based support for the biological relevance of the predominant ULK1 phosphosites.

### 2.2. Co-Occurrence Patterns Among ULK1 Phosphosites

Proteins often contain multiple phosphosites, which function independently or together to regulate protein activity and signal outputs. A large-scale phosphoproteomic study by Li et al. (2017) demonstrated that phosphosites that frequently co-occur across different biological conditions are often functionally associated, suggesting that phosphosite co-occurrence analysis is a valuable approach for understanding how phospho-signaling networks regulate cellular processes [14]. To interpret potential coordinated phosphosignaling patterns within ULK1, we then calculated the frequency of positive and negative co-occurrence patterns for each phosphosite pair within ULK1 from cellular differential datasets that quantified at least two ULK1 phosphosites together, as illustrated in Figure 2.

As mass spectrometry primarily quantifies phosphorylation at the level of tryptic peptides, we treated phosphosite pairs within the same tryptic peptide with caution, as their observed co-occurrence may partly reflect peptide-level quantification rather than independent, biologically distinct phosphorylation events [15]. In our analysis, the same-peptide phosphosite pairs (S477–S479, appearing in 26 datasets, and S467–S469, in 13 datasets) showed positive co-occurrence; however, because their apparent co-occurrence may simply reflect how mass spectrometry measures peptides, rather than genuine biological co-regulation, they were not included in further functional analysis. Among phosphosite pairs from distinct peptide regions, S623–S638, S450–S477, and S450–S469 exhibited positive co-occurrence in 11, 11 and 10 datasets, respectively. The ULK1 phosphosite co-occurrence frequencies are provided in Supplementary Materials File S2. Together, these patterns suggest coordinated phosphoregulation of ULK1 phosphosites, with different peptide pairs providing more suggestive evidence of functional association. However, further experimental studies are required to validate the biological significance of these coordinated signaling patterns.

### 2.3. Functional Characterization and Enrichment Analysis of Proteins Co-Regulated with Predominant ULK1 Phosphosites

Among the three predominant ULK1 phosphosites identified in this study (S450, S623, and S638), S638 is the most extensively characterized and has been associated with the regulation of ULK1 enzymatic activity, autophagy, apoptosis, and carcinogenesis. Furthermore, S638 undergoes starvation-induced dephosphorylation, consistent with its role as a nutrient-responsive regulatory phosphosite [16,17,18]. Similarly, S623 was identified in large-scale phosphoproteomic profiling of EGFR-driven lung cancer models and shows dynamic regulation under growth factor-dependent conditions, but its direct functional role remains unclear [19]. In contrast, limited information is currently available regarding S450’s functional role, underscoring the need for further investigation into its role in ULK1 regulation. We revised the differential datasets to identify factors that could account for the overrepresentation of phosphosites in the assembled datasets. Accordingly, we assessed the number of phosphosites in each dataset, the number of datasets within and across different research articles that investigated specific stimuli in one or multiple cell lines, including temporal responses to stimulation. The cumulative distribution of positively and negatively co-regulated proteins associated with the predominant ULK1 phosphosites across multiple datasets is shown in Figure 3. We analyzed phosphosites in phosphoproteins (PPs) that were co-regulated with predominant ULK1 phosphosites to identify potential functional relationships. ULK1 phosphosites S450, S623, and S638 were associated with 1583, 432, and 280 PPs that showed positively co-regulated proteins, respectively, whereas 15 PPs exhibited negatively co-regulated proteins with S638 phosphosites. The complete co-regulation dataset is available in Supplementary Materials File S3. The top 20 high-confidence phosphosites in phosphoproteins (PPs) co-regulated with the predominant ULK1 phosphosites are shown in Figure 4 [20].

As ULK1 is a key regulator of autophagy, we examined the biological processes and pathways associated with the positively co-regulated proteins of the predominant ULK1 phosphosites S450, S623 and S638, to assess whether phosphosite-specific co-regulation patterns are consistent with the known role of ULK1 in regulating autophagy. Gene Ontology and KEGG pathway enrichment analyses identified significant enrichment of cellular energy homeostasis pathways and autophagy regulation. The co-regulated proteins associated with S450 were enriched in the mTOR, FoxO, insulin signaling, and longevity-regulating pathways. They were also enriched for biological processes related to the positive and negative regulation of autophagy, including the negative regulation of macroautophagy. In contrast, the co-regulated proteins of S623 showed enrichment primarily in the autophagy-animal, AMPK signaling, insulin signaling, and the longevity-regulating pathways. These proteins were additionally associated with biological processes related to autophagy, macroautophagy, and other autophagy-associated functions. Similarly, the co-regulated proteins associated with S638 were enriched in the autophagy-animal and AMPK signaling pathways, together with biological processes involving autophagy, macroautophagy, and the regulation of autophagy. These biological processes and pathways are directly linked to ULK1 activity, which indicates the central role of ULK1 in regulating cellular energy sensing and autophagic processes. Furthermore, several biological processes known to support autophagosome biogenesis and autophagic flux were also significantly enriched across the three phosphosites, including components of the ubiquitin/deubiquitination system, membrane trafficking and vesicle transport, small GTPase signaling, and cytoskeletal dynamics. The complete results of the gene enrichment analysis are provided in Supplementary Materials File S4.

The top co-regulated proteins of predominant phosphosites, including SMCR8, PDPK1, SNAP23, USP7, and TRIM25 are involved in this autophagy process. To further explore the functional relevance of these associations, we examined site-specific phosphoproteomic evidence for proteins involved in autophagy. Among the proteins associated with ULK1 S450, AKT1S1 (S202 and S203) and ULK1 (S469); with ULK1 S623, STAT3 (S727) and ULK1 (S638); and with ULK1 S638, PDPK1 (S241) have been implicated in the inhibition of autophagy, whereas DAP (S3) has been reported to regulate autophagy. These results indicate that phosphorylation at the predominant ULK1 phosphosites may be linked to molecular networks involved in multiple stages of autophagy, from initiation to vesicle trafficking and autophagosome maturation, as well as in the positive and negative regulation of autophagic activity.

### 2.4. Computational Prediction of Upstream Kinases for the Predominant ULK1 Phosphosites

Protein phosphorylation is an important cellular regulatory mechanism, as many enzymes and receptors are regulated by phosphorylation and dephosphorylation mediated by kinases and phosphatases [21]. Furthermore, understanding kinase-substrate relationships is important for downstream analyses, as it helps connect phosphorylation events to specific signaling pathways, biological functions, and regulatory processes [22]. We identified computationally predicted upstream kinases for the positively co-regulated predominant ULK1 phosphosites using two independent prediction approaches. Using the NetworKIN and AKID algorithms, kinase-substrate, kinase-substrate relationships were predicted based on sequence motifs and protein interaction context (NetworKIN) and kinase domain specificity and phosphosite-surrounding peptide sequences using a deep neural network (AKID), rather than direct biochemical evidence. PAK2 (S58) and MAPK7 (T733) were predicted as upstream kinases of ULK1 phosphosites S638 and S450, respectively. All kinases reported here are computational predictions and have not been experimentally validated. In addition, using the kinome-wide substrate specificity atlas, Johnson et al. (2023) [23] predicted SNRK (S390) as an upstream kinase for S638, CDK12 (S332 and T514) and CDK13 (S400) as upstream kinases for S623, and MAPK7 (T733) as an upstream kinase for S450. The upstream kinases associated with the predominant ULK1 phosphosites are shown in Figure 5, with the corresponding data provided in Supplementary Materials File S5.

#### Downstream Substrates of the Predominant ULK1 Phosphosites

Among the positively co-regulated phosphosites, SMCR8 (S492) was identified as an experimentally validated ULK1 substrate associated with the predominant ULK1 phosphosite S638. SMCR8, along with C9ORF72 and WDR41, forms a complex that interacts with ULK1 and acts as a negative regulator of autophagy initiation. Under starvation conditions, this interaction is enhanced, whereas SMCR8 depletion increases ULK1 kinase activity and expression [24]. Despite these findings, the functional significance of ULK1-mediated phosphorylation of SMCR8 remains unclear. In addition to the experimentally validated substrate, downstream ULK1 substrates were predicted by Johnson et al. (2023) [23] using the kinome-wide substrate specificity atlas. This resource predicts kinase-substrate relationships by comparing phosphorylation site sequences with the ULK1 position-specific scoring matrix (PSSM), derived from positional scanning peptide array (PSPA) profiling of purified ULK1 against a synthetic peptide library. Candidate substrates are ranked by percentile score relative to the reference human Ser/Thr phosphoproteome, providing computational predictions rather than direct biochemical evidence. Using this approach, 66, 17, and 16 downstream substrates were predicted for the positively co-regulated ULK1 phosphosites S450, S623, and S638, respectively. The downstream substrates associated with the predominant ULK1 phosphosites are shown in Figure 5, with the corresponding data provided in Supplementary Materials File S5.

### 2.5. Protein-Normalized Cancer-Associated Dysregulation of the Predominant ULK1 Phosphosites

The three predominant ULK1 phosphosites (S450, S623, and S638) were assessed for cancer association using cProSite, comparing ULK1 protein-normalized phosphorylation levels (phosphorylation site level/total ULK1 protein abundance) between tumor and normal tissues across the 12 available cancer types: GBM, BRCA, COAD, HNSCC, KIRC, LIHC, LUAD, LSCC, OV, PDAC, STAD, and UCEC. Protein-normalized data for the three sites were obtained for 19 of the 36 possible site-cancer combinations. Among the three phosphosites, S450 was analyzed in seven cancer types (GBM, HNSCC, KIRC, LIHC, LUAD, LSCC, and UCEC), S623 in eight cancer types (GBM, HNSCC, KIRC, LIHC, LUAD, LSCC, STAD, and UCEC), and S638 in four cancer types (GBM, HNSCC, STAD, and UCEC). Protein-normalized analysis could not be performed for COAD, OV, and PDAC due to the unavailability of sufficient ULK1 protein abundance data in cProSite for all three phosphosites. Furthermore, BRCA lacked matched normal tissue data for S623 and S638; therefore, these phosphosites could not be evaluated in BRCA.

Statistical significance was defined as *p* < 0.05, and the direction of phosphorylation change was classified as upregulated for log_2_ fold-change values greater than zero and downregulated for values less than zero. At S450, phosphorylation was significantly upregulated in head and neck cancer (log_2_FC = 0.55, *p* < 0.0001), kidney cancer (log_2_FC = 0.42, *p* = 0.0016), liver cancer (log_2_FC = 0.66, *p* < 0.0001), lung adenocarcinoma (log_2_FC = 1.07, *p* < 0.0001), lung squamous cell carcinoma (log_2_FC = 0.43, *p* < 0.0001), and brain cancer (log_2_FC = 0.88, *p* < 0.0001). No statistically significant change was observed in uterine cancer (log_2_FC = 1.01, *p* = 0.1592). At S623, phosphorylation was significantly upregulated in head and neck cancer (log_2_FC = 0.42, *p* = 0.0002), kidney cancer (log_2_FC = 0.76, *p* < 0.0001), liver cancer (log_2_FC = 0.53, *p* = 0.0001), lung adenocarcinoma (log_2_FC = 0.39, *p* = 0.0005), lung squamous cell carcinoma (log_2_FC = 0.58, *p* < 0.0001), uterine cancer (log_2_FC = 0.78, *p* = 0.0078), and brain cancer (log_2_FC = 1.13, *p* < 0.0001). No statistically significant change was observed in stomach cancer (log_2_FC = 0.15, *p* = 0.4617). At S638, phosphorylation was significantly upregulated in head and neck cancer (log_2_FC = 0.93, *p* < 0.0001), uterine cancer (log_2_FC = 1.95, *p* = 0.0299), and brain cancer (log_2_FC = 3.43, *p* = 0.043). No statistically significant change was observed in stomach cancer (log_2_FC = 0.22, *p* = 0.4711). Protein-normalized phosphorylation levels of the predominant ULK1 phosphosites across multiple cancer types are shown in Figure 6.

Overall, across the 19 evaluable site-cancer comparisons, 16 showed statistically significant results. S450 exhibited significant phosphorylation changes in six of seven evaluable cancer types, S623 showed significant upregulation in seven of eight evaluable cancer types, and S638 showed significant upregulation in three of four evaluable cancer types, with S450 and S623 as the most frequently altered phosphosites. The complete protein-normalized cancer-specific phosphorylation data are provided in Supplementary Materials File S6.

### 2.6. ULK1-Interacting Proteins Suggest a Functional Network Linking Ubiquitin-Dependent Cargo Recognition with Autophagosome Biogenesis, Trafficking, and Maturation

Functional annotations of high-confidence ULK1-interacting proteins revealed an enrichment of ubiquitin-regulatory proteins that link ubiquitin signaling to selective autophagy. E3 ubiquitin ligases regulate protein homeostasis by attaching ubiquitin to specific proteins, which controls their recognition and degradation by the ubiquitin-proteasome system (UPS) or selective autophagy [25]. The identification of several E3 ligases (TRIM32, TRIM25, NEDD4L) alongside deubiquitinases (USP10, USP20, USP1 with cofactor WDR48/UAF1) within the ULK1 interactome is associated with a balance of ubiquitination and deubiquitination in this network. This ubiquitin-regulatory module is further connected to selective autophagy receptors such as OPTN (optineurin), which recognizes ubiquitinated cargo through its ubiquitin-binding domain while simultaneously binding LC3 on the developing autophagosomal membrane, linking ubiquitinated substrates to the autophagic degradation pathway [26]. Following cargo recognition, the ULK1 complex initiates phagophore assembly together with RB1CC1 (FIP200) and AMBRA1. Consistent with this role, ATG9A and ATG16L1 are involved in autophagosome biogenesis. Several interactors were associated with downstream autophagosome trafficking and maturation, including VIM, HDAC6, TBC1D5 and SMCR8. Overall, the ULK1 interactome is linked to a network that connects ubiquitin-dependent cargo recognition to autophagosome biogenesis, trafficking, maturation, and lysosomal degradation, supporting a central role for ULK1 in selective autophagy and proteostasis. The ULK1-interacting protein network is shown in Figure 7, with the corresponding interaction data provided in Supplementary Materials File S7.

### 2.7. Co-Regulated Proteins of Predominant ULK1 Phosphosites with Metabolic Enzymes Indicate Functional Associations with Metabolic Pathways

ULK1 acts as a central regulator of autophagy and plays a key role in cellular metabolic adaptation in response to nutrient and energy stress [27]. We explored whether co-regulated proteins of predominant ULK1 phosphosites were associated with metabolic pathways. We identified 13 metabolic enzymes co-regulated with ULK1 S450, seven with S623 and five with S638. These enzymes are involved in diverse metabolic pathways, including glycogen metabolism, sphingolipid metabolism, pyrimidine and phosphatidylcholine biosynthesis, coenzyme A biosynthesis, phosphoinositide signaling, glycolysis, and lipid mobilization.

Among these, four enzymes have phosphosite-specific annotations relevant to metabolic regulation, including GYS1 (S727), SPHK2 (S387), PCYT1A (S323), and PFKL (S775), two enzymes, including PANK2 (S189) and LIPE (S853, S855) are predicted substrates of ULK1 according to Johnson et al. (2023) [23]. LIPE (hormone-sensitive lipase) is a key enzyme that regulates hormone-stimulated lipolysis. Phosphorylation at S853 and S855 (human), corresponding to S552 and S554 in human isoform 2 (UniProt Q05469), represents the basal phosphorylation site of LIPE and is distinct from the major PKA-regulated site (Ser649/Ser650) [28]. Although phosphorylation at the basal site has been reported to have a relatively minor effect on lipolytic activity compared with the regulatory site. Identifying LIPE among the proteins co-regulated with the predominant ULK1 phosphosites is important because ULK1-dependent autophagy and LIPE-mediated lipolysis both contribute to lipid turnover during metabolic stress. Lipophagy promotes the lysosomal degradation of lipid droplets, whereas LIPE hydrolyzes stored neutral lipids to release free fatty acids, thereby supporting metabolic adaptation and cellular energy homeostasis [29]. Collectively, these findings suggest that ULK1 may influence metabolic processes beyond its established role in autophagy, linking its phosphosite-associated regulatory network to pathways involved in energy homeostasis, lipid utilization, and nutrient adaptation.

### 2.8. Identification of ULK1-Regulated Phosphoproteins Through Comparative Phosphoproteomic Analysis

To determine whether proteins co-regulated with the predominant ULK1 phosphosites (S450, S623, and S638) are associated with ULK1-dependent phosphorylation, we compared our co-regulated protein list with an independent, published SILAC-based phosphoproteomic dataset generated from ULK1/ULK2 double-knockout (DKO) mouse embryonic fibroblasts (MEFs) and DKO MEFs reconstituted with FLAG-tagged human ULK1 under starvation conditions as reported by Hu et al. (2021) [30]. In the original study, ULK1-dependent phosphosites were identified using a random-effects model that accounted for variability among biological replicates, phosphosites, and the number of replicates per site (*p* < 0.05), together with Significance A outlier analysis implemented in Perseus (Benjamini–Hochberg corrected, *p* < 0.05). Only phosphosites meeting both significance criteria were considered ULK1 regulated, with the direction of regulation (upregulated or downregulated in ULK1/ULK2 DKO cells relative to ULK1-reconstituted cells) determined by the sign of the estimated effect. As the phosphoproteomic dataset was generated in mouse cells, significantly regulated phosphosites were mapped to their corresponding human orthologous proteins and conserved phosphosite positions. Only phosphosites with confirmed human orthologs at conserved positions were retained for comparison with proteins co-regulated with the predominant ULK1 phosphosites identified in our phosphoproteomic analysis. For ULK1 S450, seven downregulated and eight upregulated phosphoproteins overlapped; for S623, five downregulated and one upregulated phosphoprotein overlapped; and for S638, two downregulated and two upregulated phosphoproteins overlapped, with the corresponding data provided in Supplementary Materials File S8. Collectively, these findings suggest that a subset of proteins co-regulated with the predominant ULK1 phosphosites also exhibit ULK1-dependent phosphorylation in an independent phosphoproteomic study, supporting the biological relevance of these co-regulated proteins. Among the overlapping proteins, NAV3 (S275), NAA30 (S190), PPP6R2 (S289), PXN (S137), and CAST (S577) have been predicted as downstream targets of ULK1, providing additional support for the relevance of these co-regulated proteins. However, further experimental validation is required to establish their functional significance and confirm direct ULK1-dependent regulation.

## 3. Discussion

In this study, we performed an integrative phosphoproteomic analysis of ULK1 across large-scale human profiling and differential datasets. We identified S450, S623 and S638 as the predominant phosphosites of ULK1 and characterized their associated co-regulatory networks. Among the three sites, S638 is the most extensively characterized, with established roles in mTORC1/AMPK-mediated regulation of autophagy, apoptosis and carcinogenesis; S623 has been linked to EGFR-driven lung cancer signaling but lacks a defined functional role; and S450 remains comparatively underexplored despite its high detection frequency. Co-occurrence analysis identified coordinated regulation among several phosphosite pairs, particularly between S623 and S638. This observation is consistent with prior reports that phosphosites that co-occur across diverse biological conditions are often functionally linked, even when regulated by different kinases [14]. This coordination is further reflected in the site-specific functional profiles of proteins co-regulated with each phosphosite. Among proteins linked to S450, AKT1S1 (S202 and S203) is phosphorylated by PKM2, which activates mTORC1 signaling and suppresses autophagy [31], while ULK1 (S469) is phosphorylated by TOPK, which reduces ULK1 activity and inhibits autophagy [32]. At S623, STAT3 (S727) showed context-dependent roles in both activating and inhibiting autophagy [17,33], whereas ULK1 (S638) consistently functioned as an inhibitory phosphosite across the studies examined [16]. In contrast, proteins associated with S638 included PDPK1 (S241), which promotes AKT-mTOR signaling and indirectly suppresses autophagy [34], and DAP (S3), whose phosphorylation is linked to autophagy regulation [35].

Further analysis of interactors co-regulated with predominant ULK1 phosphosites showed that ULK1 is part of a coordinated interaction network linking ubiquitin-dependent cargo recognition to autophagosome biogenesis, trafficking, and maturation. Within the ubiquitin regulatory module, TRIM32 promotes autophagy initiation by enhancing ULK1 kinase activity through unanchored K63-linked polyubiquitination [36], TRIM25 suppresses autophagy by impairing p62 function via K63-linked ubiquitination that disrupts p62 oligomerization and LC3 recruitment [37], and NEDD4L negatively regulates ULK1 stability during prolonged starvation [38], while USP10, USP20, and the USP1-WDR48 complex act as counteracting deubiquitination mechanisms within the autophagy regulatory network [39,40,41]. This ubiquitin-tagged cargo is recognized by OPTN, which bridges ubiquitinated substrates to the forming autophagosome via LC3 [26]. Several interactors were associated with downstream autophagosomes. ATG9A provides membrane sources for phagophore expansion through its lipid scramblase activity [42], while ATG16L1 promotes LC3 lipidation needed for membrane elongation [43]. Autophagosome trafficking and maturation are further supported by VIM, which helps position autophagosomes along intermediate filaments and promotes their fusion with lysosomes [44], HDAC6, which drives the clearance of ubiquitinated cargo towards aggresomes [45], and TBC1D5 and SMCR8, which regulate retromer- and Rab-GTPase-dependent maturation of autophagosomes before they fuse with lysosomes [46,47]. This network suggests that ULK1 phosphoregulation contributes to multiple stages of selective autophagy beyond its well-established role in autophagy initiation.

Notably, ULK1 phosphosites showed strong associations with metabolic enzymes spanning glycogen, sphingolipid, phospholipid, and glycolytic metabolism. GYS1 is the rate-limiting enzyme in glycogen synthesis [48]. SPHK2 phosphorylates sphingosine to produce sphingosine-1-phosphate, and ERK1/2 phosphorylation at S351 (S387 in the canonical isoform) enhances SPHK2 activation [49]. PCYT1A (CCTα) catalyzes the rate-limiting step in phosphatidylcholine biosynthesis through the CDP-choline pathway, and S323 is part of an AKT2-regulated phosphorylation cluster (S315/S319/S323) associated with enhanced stromal phosphatidylcholine production [50,51]. PFKL catalyzes the rate-limiting phosphofructokinase step in glycolysis, and phosphorylation at S775 enhances PFKL activity, which promotes glycolytic flux during innate immune activation and metabolic stress [52].

PANK2 catalyzes the first and rate-limiting step of coenzyme A (CoA) biosynthesis by phosphorylating pantothenate, thereby regulating CoA availability for key metabolic pathways such as fatty acid oxidation and the tricarboxylic acid (TCA) cycle [53]. The co-regulation of LIPE with ULK1 suggests that ULK1-dependent autophagy and classical PKA-driven lipolysis may operate in parallel during metabolic stress. This finding supports an association between these pathways rather than direct regulatory interaction between them. Together, the phosphosite-specific associations suggest that ULK1 phosphoregulation may have a broader role in coordinating cellular energy metabolism, consistent with its established role in metabolic adaptation [27].

Protein-normalized cancer-associated analysis revealed site-specific and tumor-specific phosphorylation patterns across the 12 available cancer cohorts. Among the predominant phosphosites, S450 and S623 showed significant upregulation in more of the evaluated cancer types than S638, indicating that S450 and S623 may be more broadly influenced by oncogenic signaling, whereas S638 dysregulation appears more restricted, though this may also partly reflect its more limited coverage in the available protein-normalized data. These results demonstrate that protein-normalized phosphosite analysis can effectively distinguish site-specific phosphorylation changes across different cancer types. Consistency with an independent ULK1/ULK2 knockout phosphoproteomic dataset strengthens the relevance of the identified co-regulated proteins and suggests that our large-scale phosphoproteomic data integration can identify candidate ULK1-regulated phosphoproteins for future mechanistic studies, although direct kinase-substrate relationships remain to be experimentally established. While these findings support a role for ULK1 activity, they are based primarily on computational analyses, including co-regulation, interaction mapping, and cross-dataset comparisons. Further experimental studies, particularly site-directed mutagenesis and biochemical assays, are needed to define the mechanistic roles of S450 and S623.

## 4. Material and Methods

### 4.1. Curation, Screening, and Assembly of Global Phosphoproteome Datasets of ULK1

The global ULK1 phosphoproteomic datasets were assembled and analyzed using a previously established pipeline developed in our laboratory [54]. Briefly, relevant datasets were identified through a comprehensive and systematic literature search of PubMed using the terms “phosphoproteomics” OR “phosphoproteome”. Only datasets based on human cell lines were included for analysis, while studies categorized as “Plant” or “Review” articles were excluded. We screened the 173 publications to assemble human cell line-based global high-throughput phosphoproteome datasets with ULK1 phosphorylation sites. These curated datasets were generated from the quantitative strategies (including label-free, TMT, and SILAC), mass spectrometry platforms (predominantly Orbitrap-based instruments), phosphopeptide enrichment approaches (including TiO and Fe-IMAC), LysC- and/or trypsin-digested phosphopeptide analyses, and criteria used for Class I phosphosite assignment are provided in Supplementary Materials File S1. Because these parameters are major contributors to technical variation in phosphoproteomic analyses, they were carefully considered during dataset selection to maintain consistency and improve comparability across the integrated studies. To further refine our analysis, we fetched the Class I ULK1 phosphorylation sites, using a localization probability of ≥75% or an A-score >13 [55].

These curated datasets were further classified into two categories: quantitative differential datasets, which compare test biological or experimental conditions against corresponding controls, and qualitative profile datasets, which consider test and control conditions as independent datasets. Phosphosites with a fold change of ≥1.3 were classified as upregulated, whereas those with fold changes ≤ 0.76 were classified as downregulated. A significance threshold of *p* < 0.05 was used to identify statistically significant changes across different biological conditions. To minimize inconsistencies in gene nomenclature, all proteins were mapped to their respective HUGO Gene Nomenclature Committee (HGNC)-approved gene symbols. To ensure consistent and standardized annotation, phosphosites were mapped to UniProt (2023) accessions using an in-house mapping tool [56].

### 4.2. Comprehensive Mapping of Predominant Phosphorylation Sites Representing ULK1

To identify the predominant phosphosites of ULK1 from the human cellular qualitative profile and quantitative differential phosphoproteomic datasets enriched for S/T/Y, phosphosites were ranked according to their frequency of detection across the compiled datasets [57]. Among them, phosphosites with the highest frequency were selected as the ULK1 predominant phosphosites, and their co-regulated phosphosites in other proteins (PPs) were subsequently determined. To obtain phosphosite-specific information, the predominant ULK1 phosphosites were analyzed using the PhosphoSitePlus database [58] and the PoKD/PiKD framework described by Gopalakrishnan et al. (2025) [13].

### 4.3. Co-Occurrence Analysis of Differentially Regulated ULK1 Phosphosites

A co-occurrence analysis was performed to examine how different phosphorylation sites in ULK1 are connected to one another, with a particular focus on identifying phosphosite pairs that share similar differential regulation patterns within ULK1 [59]. We selected differentially regulated datasets containing multiple ULK1 phosphosites identified under the same experimental conditions for co-occurrence analysis. For each pair of phosphosites, the frequencies of the four possible regulation combinations (UU, UD, DD, and DU) were determined. Positive co-regulation was calculated as the ratio of phosphosite pairs regulated in the same direction (UU and DD) to those regulated in opposite directions (UD and DU). Negative co-regulation was calculated as the ratio of phosphosite pairs regulated in opposite directions (UD and DU) to those regulated in the same direction (UU and DD) [60]. Specifically, the ratio [Σ(nUU + nDD)/Σ(nUD + nDU)] was applied to evaluate positive co-regulation and [Σ(nUD + nDU)/Σ(nUU + nDD)] to evaluate negative co-regulation, with higher ratio values interpreted as indicating stronger co-regulation between a given phosphosite pair. These co-occurrence patterns were used to assess potential functional associations between ULK1 phosphosite pairs.

### 4.4. Analysis of Co-Regulated Phosphosites in Other Proteins (PPs) with ULK1 Predominant Phosphosites

To characterize co-regulatory relationships, ULK1 phosphosites were compared with phosphosites identified in other proteins (PPs) that show positive or negative co-regulation with ULK1 predominant phosphosites across multiple differential phosphoproteomic datasets. Based on their relative regulation patterns, phosphosite pairs were assigned to one of four categories: UU, DD, UD, or DU. In these categories, the first part denotes the regulation status of the ULK1 phosphosite, while the second part denotes the regulation status of the corresponding phosphosite in another protein. Here, “U” represents upregulation and “D” represents downregulation. These four categories were classified into two broader groups. Phosphosites from other proteins that were regulated in the same direction, either both upregulated or both downregulated (UU and DD), were classified as positively co-regulated phosphosites (UUDD). Pairs regulated in opposite directions (UD and DU) were classified as negatively co-regulated phosphosites (UDDU) [61].

To evaluate the significance of co-regulation between ULK1 predominant phosphosites and their associated PPs across quantitative differential datasets, we performed Fisher’s exact test (FET) using contingency table analysis and the following formula:

$$\sum p=\frac{\left(a+b)!(c+d)!(a+c)!(b+d\right)}{n!}\;\sum_{i}\frac{1}{a!b!c!d!}$$

Each phosphosite pair was represented as a 2 × 2 contingency table summarizing four possible outcomes across experimental conditions. “*a*” represents experimental conditions in which neither phosphosite was differentially regulated, while “*b*” corresponds to conditions where only one of the two phosphosites was differentially regulated, irrespective of the direction of regulation (up- or downregulation). The category “*c*” denotes pairs of phosphosites that show opposite regulation patterns (differential negative regulation), while “*d*” represents pairs that exhibit the same direction of regulation (differential positive regulation). To reduce potential bias in the observed co-regulation patterns, we applied inclusion criteria to the FET-derived protein phosphosites, including (I) FET criteria for co-regulation, which include a score with a *p*-value < 0.05; (II) FDR (false discovery rate) < 0.05; (III) a frequency cutoff of ≥10% from the total differential frequency of the predominant site for positive or negative co-regulation; (IV) consistent co-regulation reported in at least three independent publications (PMID confidence); and (V) observation across a minimum of three distinct experimental conditions (code count). Phosphosite pairs satisfying all five criteria were retained as phosphosites in other proteins (PPs) positively co-regulated (UUDD) or negatively co-regulated (UDDU) with the predominant ULK1 phosphosites [62].

### 4.5. Mapping of Protein-Protein Interactors of ULK1 and Their Co-Regulated Phosphosites

To build an extensive protein interaction landscape for ULK1, we incorporated both experimentally reported and computationally predicted protein–protein interactions that were retrieved and integrated from several well-established curated databases, including the Human Protein Reference Database (HPRD) [63], the Biomolecular Interaction Network Database (BIND) [64], BioGRID (version 4.1.190) [65], ConsensusPathDB (version 35) [66] and RegPhos 2.0 [67]. From these sources, phosphosites of ULK1-interacting proteins were cross-referenced with the high-confidence co-regulated phosphosites identified in our analysis.

### 4.6. Identification of Known and Predicted Upstream Kinases and Downstream Substrates of ULK1

To characterize the upstream and downstream regulatory mechanisms of the ULK1 interaction landscape, both experimentally validated and computationally predicted kinase-substrate relationships were systematically gathered. Experimentally confirmed upstream kinases and downstream substrates of ULK1 were retrieved from PhosphoSitePlus (v6.8.3) [68], Phospho.ELM (v7.0) [69] and RegPhos 2.0 [67]. To complement the curated annotations, we also included computational predictions from NetworKIN (accessed on 4 January 2023) [70], the Automatic Kinase-specific Interactions Detection tool (AKID) (accessed on 24 May 2023) [71], and the in vitro Kinase-to-Phosphosite Database (iKiP-DB) (v1.6.10.43) [72]. In addition, we included kinase-substrate relationships from phosphomotif analysis by Johnson et al. (2023) [23], which was based on positional scanning peptide array (PSPA) profiling of the human serine/threonine kinome, using a strict cutoff above the 90th percentile. Phosphosites associated with predicted upstream kinases and downstream substrates that also showed co-regulation with predominant ULK1 phosphosites were prioritized. This co-regulatory pattern was further used as an additional layer of confidence to support biologically meaningful kinase-substrate relationships.

### 4.7. Data Analysis and Visualization of the ULK1 Phosphoproteome

Phosphorylation data were visualized using multiple tools to generate informative and well-structured representations of the results. Phosphosite frequencies across qualitative and quantitative datasets were computed using the R/Bioconductor package trackViewer (v1.47.0) [73] and visualized as lollipop plots. Cumulative distribution plots of phosphosites were created using Python’s Matplotlib (v3.10.0) [74] and Pandas libraries (v2.3.3) [75]. Multiple sequence alignment was performed using Clustal Omega (v1.2.4) to assess the evolutionary conservation of the predominant ULK1 phosphosites and to compare their conservation across human ULK family members and mammalian species [76]. Gene Ontology (GO) and KEGG pathway enrichment analyses of the positively co-regulated proteins associated with the predominant ULK1 phosphosites were performed using the DAVID Bioinformatics Resource (2021 update) [77]. Enrichment was assessed using DAVID’s default EASE score, a modified Fisher’s exact test. Terms with a nominal *p*-value < 0.05 were considered enriched. No correction for multiple testing was applied; therefore, these enrichment results were interpreted as exploratory and hypothesis-generating. Cancer-specific phosphosite analysis was performed using cProSite, an interactive web platform for proteomics, phosphoproteomics, and genomics data analysis [78]. Further visualization and pathway mapping were performed using Cytoscape (v3.10.4), Adobe Illustrator (2020) [79], RAWGraphs 2.0 [80] and BioRender (2023) [81] for graphical representation and network-based analysis.

## 5. Conclusions

This study provides an integrative phosphoproteomic characterization of ULK1, identifying S450, S623 and S638 as its predominant phosphosites and providing insights into their co-regulatory networks, interacting proteins, upstream kinases, and downstream substrates. The conservation of these phosphosites across mammalian species, together with their differential conservation among human ULK family members, further supports their potential biological significance. Together, these findings indicate that ULK1 phosphoregulation is associated with processes beyond autophagy initiation, including ubiquitin-dependent selective autophagy, vesicle trafficking, and metabolic adaptation. The association with LIPE S855 further suggests a potential role for ULK1 phosphoregulation in lipid metabolism alongside its well-established function in lipophagy. Cancer-associated analysis further revealed site-specific phosphorylation alterations across multiple cancers, with S450 and S623 showing the most widespread alterations, suggesting that ULK1 phosphoregulations may be relevant to cancer-associated signaling. Comparative analysis with an independent ULK1/2 knockout phosphoproteomic dataset further supported the relevance of these identified co-regulated protein phosphosites. Collectively, these findings highlight candidate phosphosites and associated proteins for future functional studies aimed at better understanding the role of ULK1 phosphoregulation in autophagy, metabolism, and cancer-associated signaling.

## 6. Limitations of the Study and Future Perspectives

Although this study provides an integrative phosphoproteomic characterization of ULK1 based on the large-scale integration and analysis of profiling and differential phosphoproteomic datasets, several limitations should be acknowledged. As the study relies on publicly available mass spectrometry datasets, the results are inherently influenced by differences in experimental design, biological context, and data quality across studies. Although rigorous criteria were applied to identify the predominant phosphosites and their co-regulated partners, phosphosite pairs located on the same tryptic peptide could not be reliably distinguished from genuine biological co-regulation because their apparent co-occurrence may simply reflect how mass spectrometry measures peptides rather than true independent phosphorylation events. These pairs were therefore excluded from the main functional interpretation, while co-regulation between phosphosites on distinct peptides was considered more informative of genuine biological relationships. Furthermore, upstream kinase assignments for S450 and S623 were derived primarily from computational predictions rather than experimental validation, and the absence of direct biochemical or cellular validation limits confidence in their functional interpretation. Nonetheless, S450, S623, and S638 emerged as key regulatory phosphosites. Future experimental studies will be essential to validate these findings and define their precise molecular roles.

## Figures and Tables

**Figure 1:**
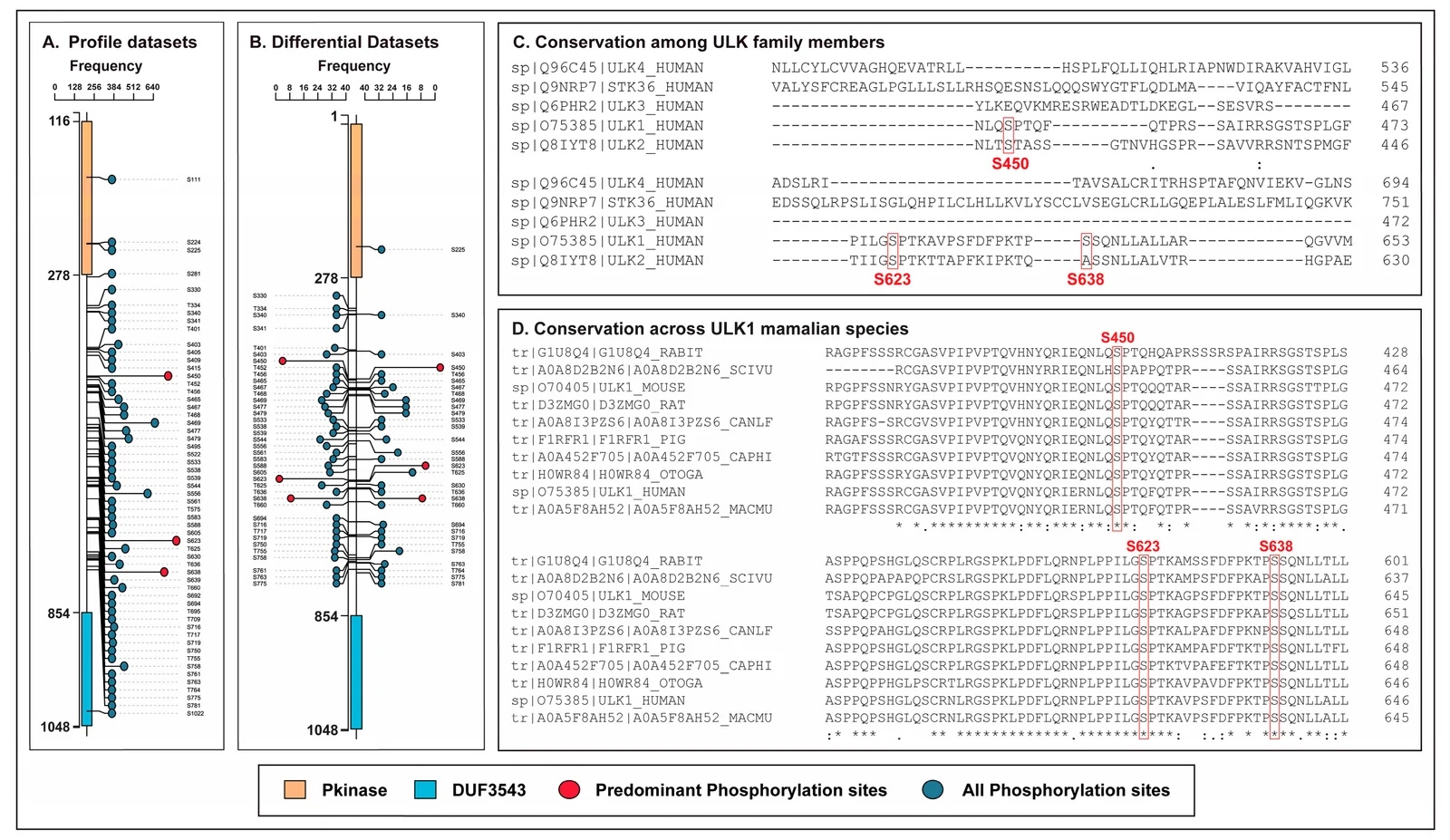
Lollipop plot of ULK1 phosphosites identified across large-scale quantitative differential and qualitative profiling phosphoproteomic datasets. (**A**) Phosphosite detection frequencies across 891 qualitative profiling datasets. (**B**) Phosphosite detection frequencies across 245 quantitative differential phosphoproteomic datasets. Orange and blue boxes denote the Pkinase and DUF3543 functional domains of ULK1, respectively. Red circles indicate the predominant ULK1 phosphosites (S450, S623, and S638), whereas blue circles represent all other detected phosphosites. (**C**) Conservation of the predominant phosphosites among human ULK family members. (**D**) Conservation of the predominant phosphosites across representative mammalian ULK1 orthologs. For panels (**C**,**D**), the red boxes indicate the predominant phosphosites of ULK1. In the sequence alignment, an asterisk (*) denotes complete conservation, a colon (:) indicates strong similarity between residue groups, and a period (.) denotes weak similarity.

**Figure 2:**
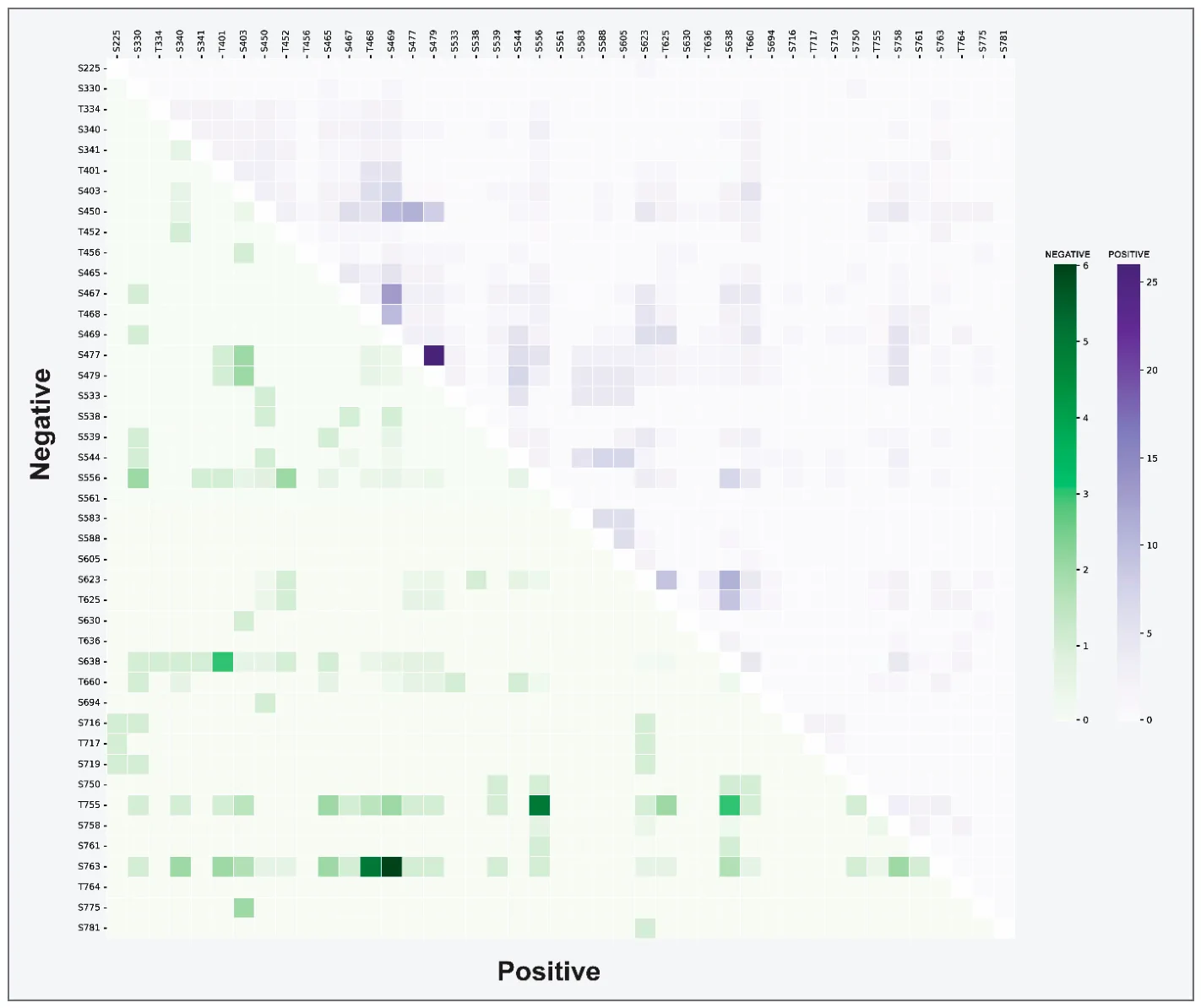
Co-occurrence of ULK1 phosphosites. Heatmap illustrates positive and negative co-regulation patterns among ULK1 phosphosites. Positive co-regulation is represented in purple and negative co-regulation in green. The intensity of the color corresponds to the frequency with which each phosphosite pair was co-regulated across the analyzed datasets.

**Figure 3:**
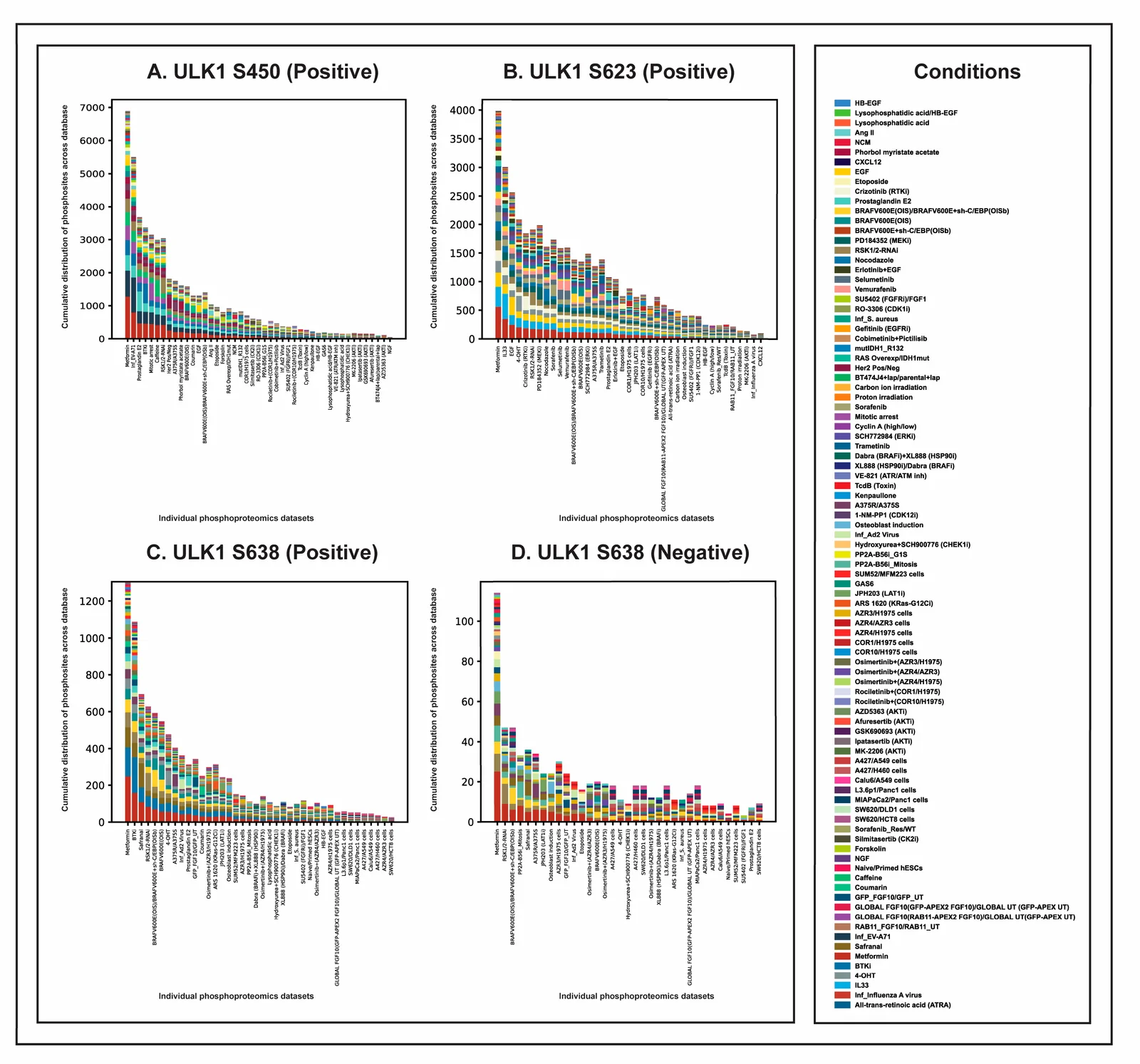
Distribution of positively and negatively co-regulated phosphosites in other proteins (PPs) associated with ULK1 predominant sites (FET *p*-value < 0.05, FDR < 0.05) across individual phosphoproteomic datasets. (**A**) Cumulative distribution of positively co-regulated phosphosites in other proteins (PPs) associated with ULK1 predominant site S450 across individual phosphoproteomic datasets. (**B**) Cumulative distribution of positively co-regulated phosphosites in other proteins (PPs) associated with ULK1 predominant phosphosite S623 across individual phosphoproteomic datasets. (**C**) Cumulative distribution of positively co-regulated phosphosites in other proteins (PPs) associated with ULK1 predominant phosphosite S638 across individual phosphoproteomic datasets. (**D**) Cumulative distribution of negatively co-regulated phosphosites in other proteins (PPs) associated with ULK1 predominant phosphosite S638 across individual phosphoproteomic datasets. Each stacked bar represents an individual phosphoproteomic dataset. The x-axis denotes individual datasets, while the y-axis represents the cumulative distribution of associated co-regulated phosphosites detected in datasets. Different colors represent experimental conditions (stimulus, treatment, or cellular system) contributing to each dataset, as specified in the conditions. The total height of each bar represents the number of associated phosphosites identified in that dataset. The presence of co-regulated phosphosites across multiple independent datasets and experimental conditions demonstrates that these associations extend across diverse experimental contexts.

**Figure 4:**
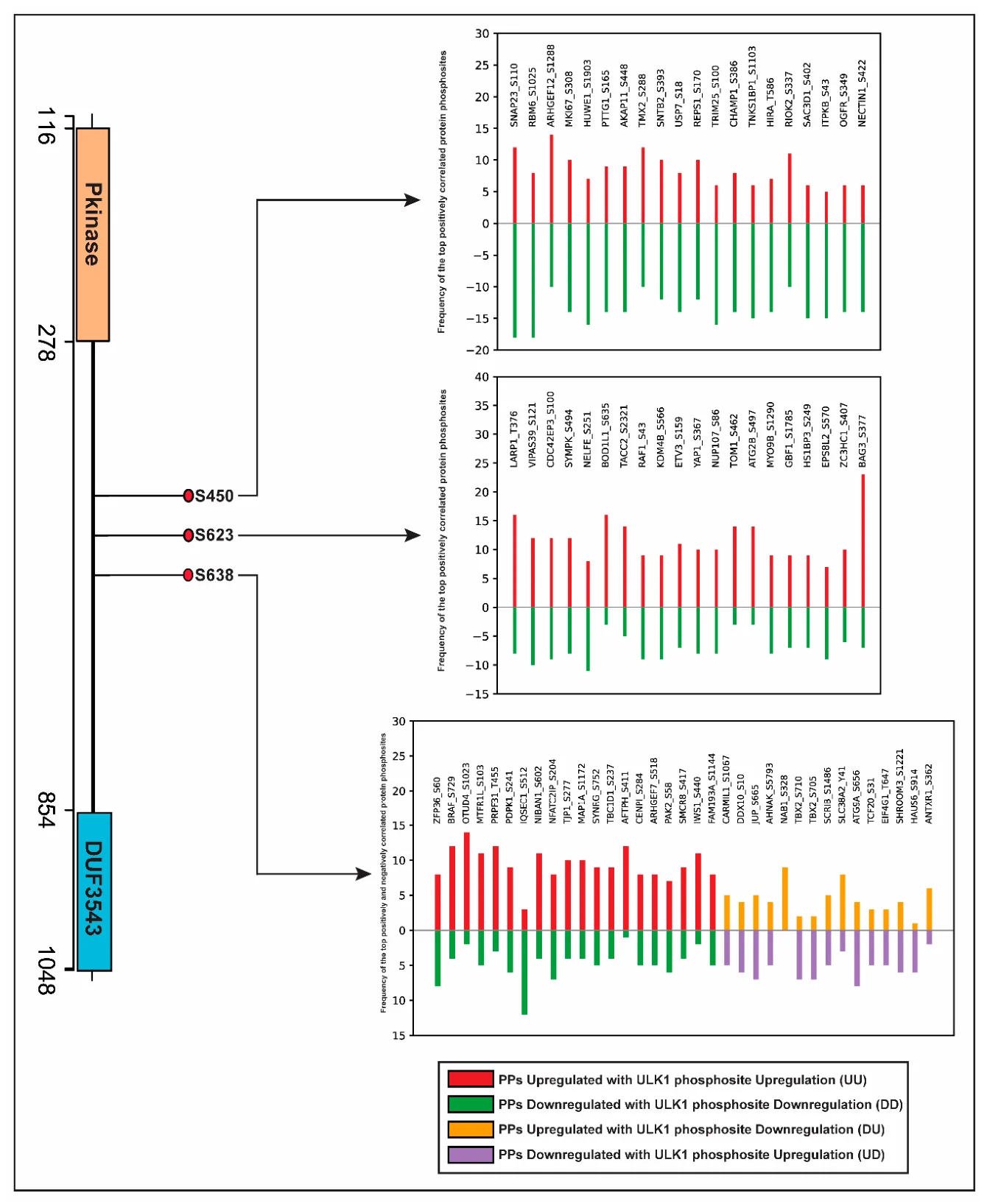
The top 20 phosphosites in other proteins (PPs) showing co-regulation with the predominant ULK1 phosphosites (S450, S623, S638). Co-regulation direction is denoted using a two-letter code, where the first letter indicates the regulation status of the ULK1 phosphosite and the second letter indicates the regulation status of the corresponding phosphosite in the co-regulated protein (“U” = upregulated, “D” = downregulated). UU and DD denote positive co-regulation (same-direction change); UD and DU denote negative co-regulation (opposite-direction change).

**Figure 5:**
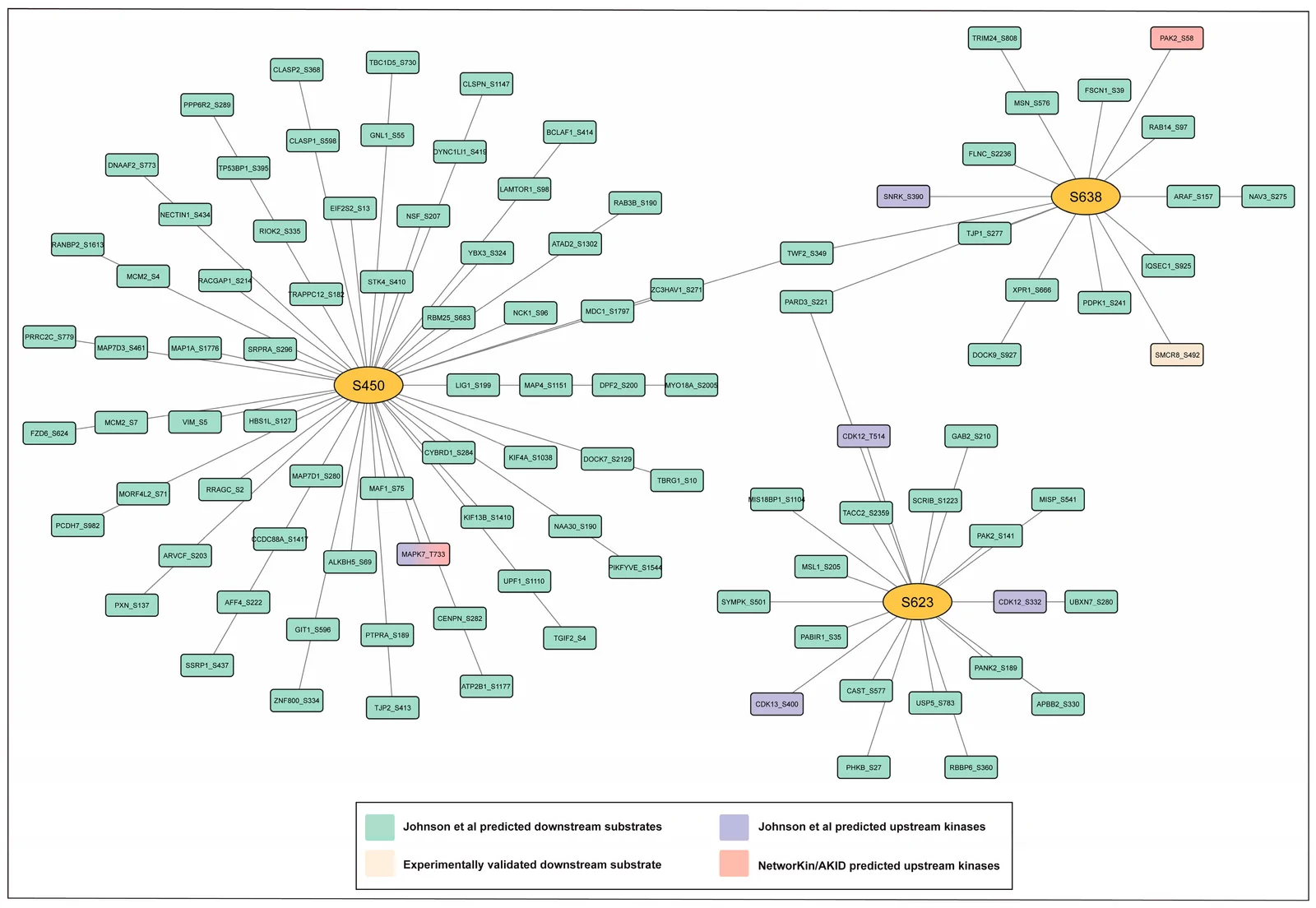
Integrated interaction network of the predominant ULK1 phosphosites (S450, S623, and S638). Yellow nodes represent the predominant ULK1 phosphosites. Green nodes denote downstream substrates reported by Johnson et al. (2023) [23], beige nodes indicate experimentally validated downstream substrates, purple nodes represent upstream kinases predicted by Johnson et al. (2023) [23], and red nodes denote upstream kinases predicted by NetworkIN and AKID. Edges represent phosphosite-specific regulatory interactions.

**Figure 6:**
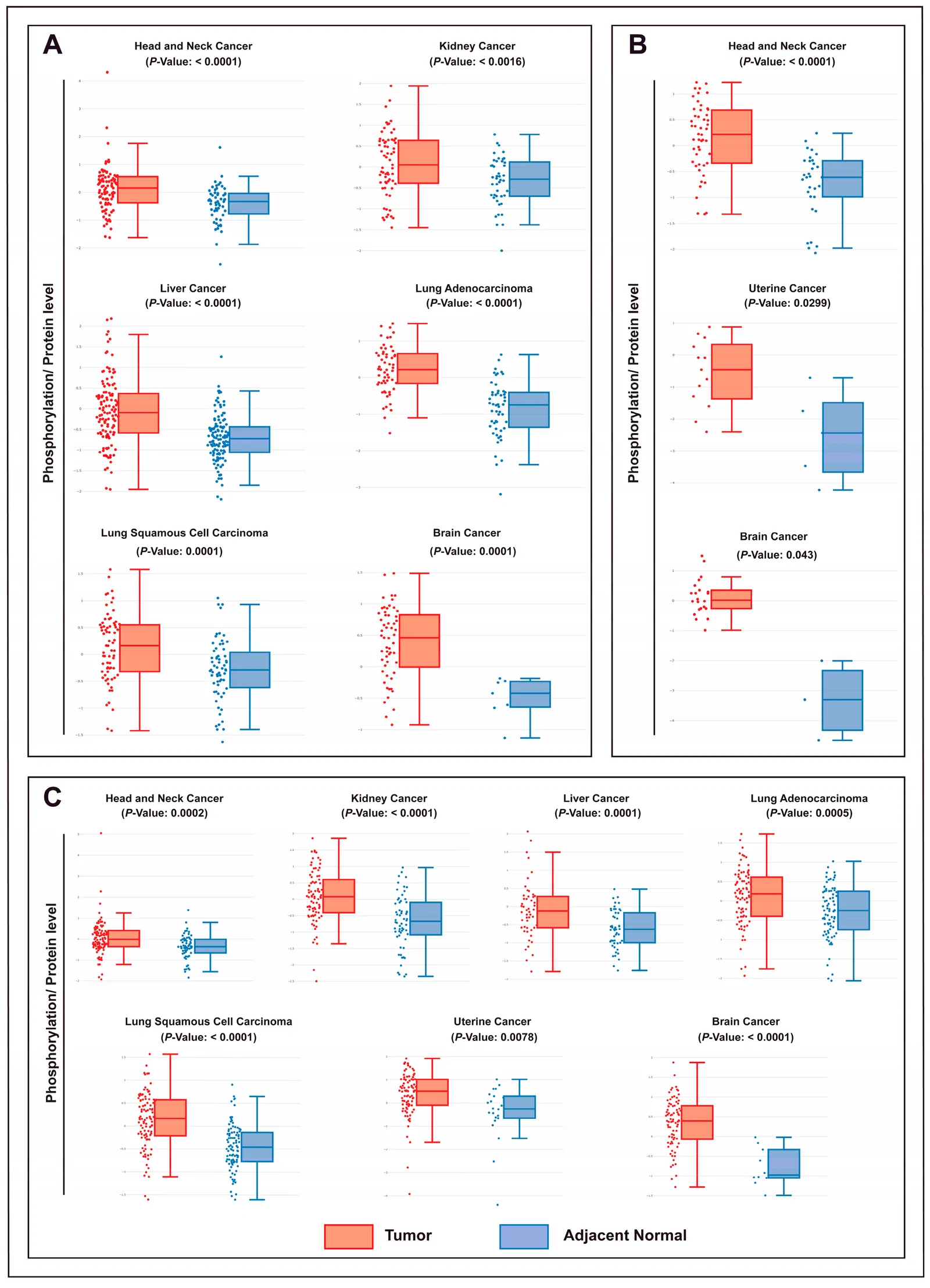
ULK1 protein-normalized phosphorylation levels of the predominant ULK1 phosphosites across cancer types with statistically significant changes. Box-and-scatter plots comparing tumor (red) and adjacent normal tissue (blue) for phosphorylation/protein level from cProSite, shown only for cancer types reaching statistical significance (*p* < 0.05). (**A**) S450, significantly upregulated in HNSCC, KIRC, LIHC, LUAD, LSCC, and GBM. (**B**) S638, significantly upregulated in HNSCC, UCEC, and GBM. (**C**) S623, significantly upregulated in HNSCC, KIRC, LIHC, LUAD, LSCC, UCEC, and GBM.

**Figure 7:**
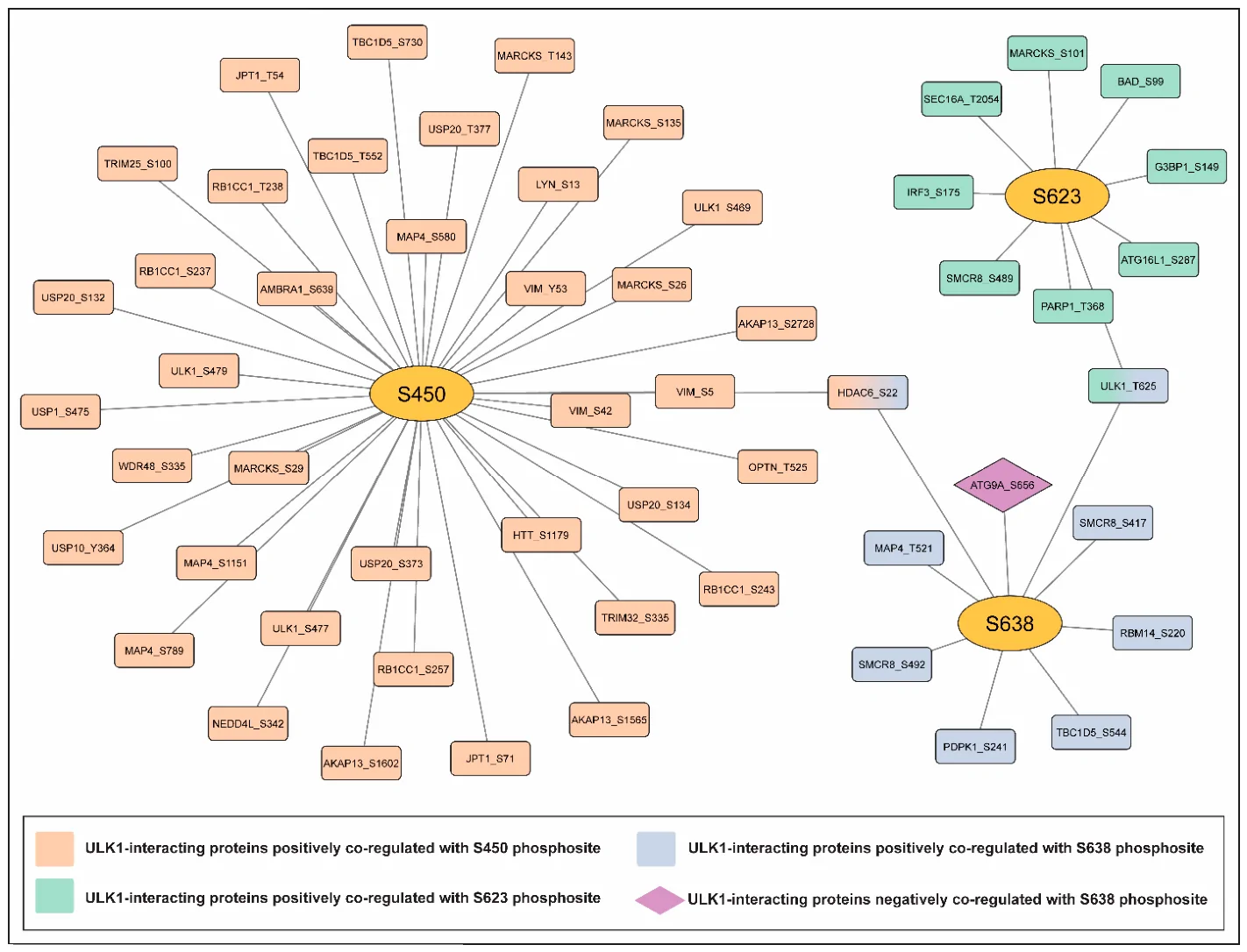
Known ULK1-interacting proteins identified from BioGRID, HPRD, BIND, and ConsensusPathDB were compared with the proteins present in the co-regulated PP list for each predominant ULK1 phosphosite to identify overlapping proteins. These associations represent phosphosite co-regulation patterns and do not imply phosphosite-specific physical interactions between ULK1 and the indicated proteins. Yellow nodes represent the predominant ULK1 phosphosites (S450, S623, and S638). Peach, green, and blue nodes denote ULK1-interacting proteins positively co-regulated with S450, S623, and S638, respectively, whereas the purple diamond represents a ULK1-interacting protein negatively co-regulated with S638. Edges represent phosphosite co-regulation patterns identified across phosphoproteomic datasets.

## Data Availability

The datasets used for the analysis are available in the supplementary file.

## Supplementary Materials

The following supporting information can be downloaded at https://www.mdpi.com/article/10.3390/ijms27198892/s1.
